# Effect of Mechanical Vibration on the Crystallization Behavior of ZBLAN Fluoride Glass Under Controlled Thermal Treatment

**DOI:** 10.3390/ma19132903

**Published:** 2026-07-06

**Authors:** Ayush Subedi, Anthony Torres, Jeff Ganley, Ujjwal Dhakal

**Affiliations:** 1 Materials, Science, Engineering and Commercialization (MSEC), Texas State University, San Marcos, TX 78666, USA; anthony.torres@txstate.edu; 2 Air Force Research Laboratory, Space Vehicles Directorate, Kirtland AFB, Albuquerque, NM 87117, USA; 3 Department of Physics, Texas State University, San Marcos, TX 78666, USA

**Keywords:** ZBLAN glass, fluoride glass, crystallization, vibration, optical attenuation, thermal-treatment

## Abstract

ZBLAN (ZrF_4_-BaF_2_-LaF_3_-AlF_3_-NaF) fluoride glass is a promising infrared optical fiber material because of its wide transmission window and low theoretical attenuation; however, unwanted crystallization during thermal processing can introduce scattering centers and degrade optical performance. Previous studies have mainly focused on temperature effects and microgravity-based crystallization suppression, while the role of mechanical vibration remains insufficiently understood. This study addresses this gap by investigating how controlled mechanical vibration influences crystallization onset, morphology, and structural evolution in ZBLAN glass during short-duration thermal treatment. ZBLAN samples were treated at selected temperatures with and without vibration using a custom heating–vibration apparatus and characterized by optical microscopy, scanning electron microscopy (SEM), energy-dispersive X-ray spectroscopy (EDS), atomic force microscopy (AFM), and X-ray diffraction (XRD). Temperature-only treatment produced a gradual transition from transparent amorphous glass to crystallized structures with increasing temperature. Vibration-assisted treatment altered crystallization behavior, producing distinct needle-like, bow-tie, and feather-like morphologies depending on temperature and vibration intensity. AFM confirmed a significant increase in surface roughness, while XRD verified structural evolution from amorphous to highly crystallized states. At higher vibration levels, irregular crystallization suggested that excessive sample movement may reduce thermal contact and change the effective heating condition. These findings demonstrate that mechanical vibration is a critical and controllable processing variable in ZBLAN fabrication and should be carefully managed to suppress unwanted crystallization in both terrestrial and space-based fiber manufacturing.

## 1. Introduction

Optical fibers are widely used in telecommunications, sensing, laser delivery, medical devices, defense systems, and mid-infrared photonic technologies. Although silica-based fibers dominate conventional optical communication because of their excellent mechanical stability and very low optical loss of approximately 0.15 dB/km at telecommunication wavelengths, their use is limited in the mid-infrared region, where attenuation can increase dramatically. For example, silica fibers can exhibit losses on the order of 800 dB/m at infrared wavelengths, limiting their suitability for mid-infrared applications. This limitation has motivated continued interest in soft-glass fiber systems, particularly fluoride glasses, which offer broader infrared transmission windows and lower phonon energies than silica [1,2,3,4,5].

Among fluoride glasses, ZBLAN (ZrF_4_-BaF_2_-LaF_3_-AlF_3_-NaF) is one of the most promising materials for infrared optical fiber fabrication because of its wide transmission window, low theoretical attenuation, and favorable glass-forming ability compared with many other fluoride glass compositions [2,3,4,5]. Recent studies further confirm the importance of ZBLAN and related fluoride fibers for modern mid-infrared photonic systems. Yu et al. demonstrated a high-power femtosecond Er: ZBLAN master-oscillator power-amplifier system, showing the potential of Er: ZBLAN fibers for compact, high-power mid-infrared laser generation [6]. Kawase et al. reported dispersion-managed mode-locked Tm: ZBLAN fiber lasers, demonstrating the importance of dispersion control in ZBLAN-based ultrafast laser operation [7]. Yang et al. achieved enhanced Raman soliton generation in Er: ZBLAN fiber with high pulse energy and peak power, highlighting the role of ZBLAN in tunable mid-infrared ultrafast sources [8]. Grebnev et al. reviewed recent progress in fluoride and chalcogenide glass fiber components, emphasizing both the progress and remaining challenges in developing reliable mid-infrared fiber laser and amplifier systems [9]. Collectively, these studies demonstrate the growing technological relevance of ZBLAN-based fluoride fibers. However, their focus is mainly on laser performance, dispersion management, nonlinear pulse generation, and component development after fiber fabrication. They do not directly address how processing conditions, particularly mechanical vibration during thermal treatment, influence ZBLAN crystallization, which remains a key limitation for reliable low-loss fiber fabrication.

A major challenge in ZBLAN processing is its tendency to crystallize during thermal treatment and fiber drawing. Crystallization introduces scattering centers, increases optical attenuation, and disrupts the amorphous structure required for low-loss infrared transmission. Previous studies have shown that ZBLAN crystallization is strongly temperature-dependent and can be reduced under microgravity conditions, where gravity-driven convection and diffusion are suppressed [10,11,12]. These studies are important because they establish the role of thermal and transport effects in ZBLAN crystallization. However, they do not systematically isolate the effect of mechanical vibration, even though vibration and g-jitter may occur in both terrestrial and space-based processing environments.

Mechanical vibration is an important but insufficiently studied processing variable for ZBLAN fiber fabrication. Vibration may influence sample stability, local heat transfer, nucleation behavior, and crystal morphology. General glass-crystallization studies suggest that mechanical vibration can affect atomic mobility, nucleation, and crystal growth [13], but this effect has not been systematically evaluated for ZBLAN under controlled short-duration thermal treatment. Although the present experiments were conducted under terrestrial 1 g conditions, this approach provides a controlled baseline for separating vibration effects from other microgravity-related variables. It is also directly relevant to ground-based ZBLAN fabrication, which remains more accessible and cost-effective than space-based manufacturing.

Therefore, this study investigates how controlled mechanical vibration influences the crystallization behavior of ZBLAN fluoride glass during short-duration thermal treatment. Temperature-only and vibration-assisted treatments are compared to determine how vibration condition affects crystallization onset, crystal morphology, surface roughness, and structural evolution. Optical microscopy, scanning electron microscopy, energy-dispersive X-ray spectroscopy, atomic force microscopy, and X-ray diffraction are used to evaluate the resulting morphological, compositional, topographic, and structural changes. By identifying mechanical vibration as a critical processing variable, this work provides guidance for suppressing unwanted crystallization during ZBLAN fiber fabrication and supports the development of more reliable terrestrial and microgravity-relevant processing strategies.

## 2. Materials and Methods

### 2.1. Experimental Apparatus and Vibration Characterization

**Figure 1:**
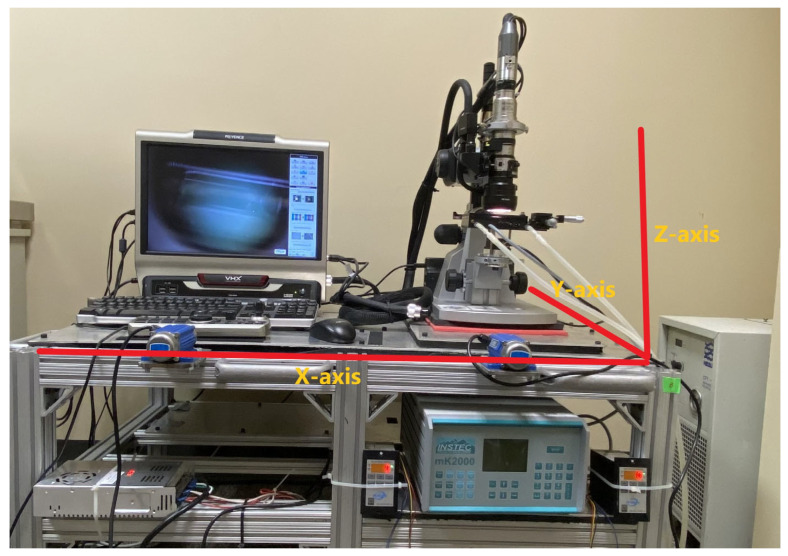
Experimental apparatus used for vibration-assisted thermal treatment of ZBLAN glass.

To investigate the effect of mechanical vibration on the crystallization behavior of ZBLAN fluoride glass, a previously developed ZBLAN processing apparatus (developed by Torres et al. [14]) was retrofitted for this study and is shown in Figure 1. The modified system consisted of an 80/20 aluminum frame equipped with a Keyence VHX-2000 digital optical microscope, an Instec heated optical viewing stage, external temperature control, a recirculating water chiller, and two independently operated vibration motors. This configuration allowed sealed ZBLAN samples to be exposed to controlled temperature and vibration conditions while enabling real-time optical observation during treatment.

The Instec heated stage provided temperature control from room temperature up to 500 °C. The stage was integrated with an external temperature controller and a recirculating water chiller unit to maintain thermal stability and reduce radiative heat accumulation around the microscope assembly. A customized ampoule holder was used to position sealed silica ampoules containing ZBLAN specimens directly on the heated stage.

Mechanical vibration was introduced using two miniature vibration motors mounted at different locations on the 80/20 frame. The low-speed motor, operating at a nominal speed of 3000 RPM, was mounted close to the heated stage, whereas the high-speed motor, operating at a nominal speed of 7000 RPM, was mounted approximately 75 cm away from the stage to generate distinct vibration conditions. The motors were operated independently to evaluate the influence of vibration frequency and intensity on ZBLAN crystallization.

The vibration response of the system was measured using a tri-axial accelerometer positioned below the heated stage at a location close enough to record stage vibration while avoiding direct heat exposure. Vibration frequencies were recorded along the X, Y, and Z axes. Each motor controller was divided into five discrete vibration levels, denoted as L1 to L5 for the low-speed motor and H1 to H5 for the high-speed motor. Each vibration level was measured for 60 s and repeated three times per day over three separate days to obtain reliable average vibration values. These measured vibration frequencies were used to correlate crystallization behavior with vibration intensity.

### 2.2. ZBLAN Sample Preparation

The ZBLAN preform material used in this study was obtained from Fiber Labs. The preform consisted of a ZBLAN core composition of 53% Zr, 27% Ba, 3% La, 3% Al, and 14% Na, and an HBLAN cladding composition of 50% Hf, 17% Ba, 5% La, 4% Al, and 24% Na. The preforms had a cladding diameter of 1.0 ± 0.1 mm and a core diameter of 0.75 ± 0.1 mm.

ZBLAN sections approximately 3–5 mm in length were cut from the same preform batch under a dry nitrogen atmosphere to minimize exposure to oxygen and moisture. Each evacuated silica ampoule contained two ZBLAN sections separated by a silica spacer with the same nominal composition and dimensions to prevent direct ZBLAN-to-ZBLAN contact during treatment. To maintain consistency across experiments, each ampoule was prepared using the same nominal configuration, consisting of one relatively longer and one relatively shorter ZBLAN section within the 3–5 mm size range. This configuration was used to maintain comparable sample loading while allowing representative sample-size variation within the manually prepared sections. Although minor variations in sample length may affect local thermal mass and contact area, all samples were processed using identical cutting, sealing, evacuation, heating, vibration, positioning, and quenching procedures. Before treatment, the target temperature and vibration condition were checked, and the ampoule was positioned as consistently as possible within the heated chamber to maintain comparable thermal exposure across the testing matrix.

**Figure 2:**
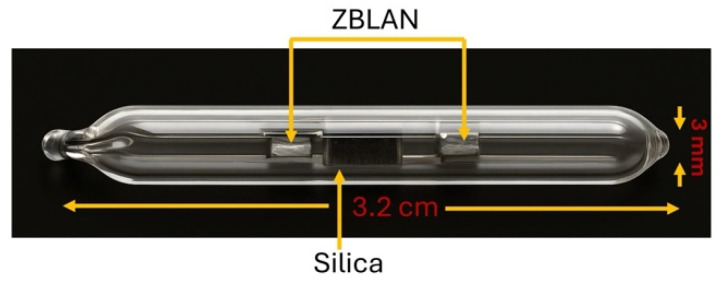
Sealed silica ampoule containing two ZBLAN glass sections separated by a silica spacer.

**Figure 3:**
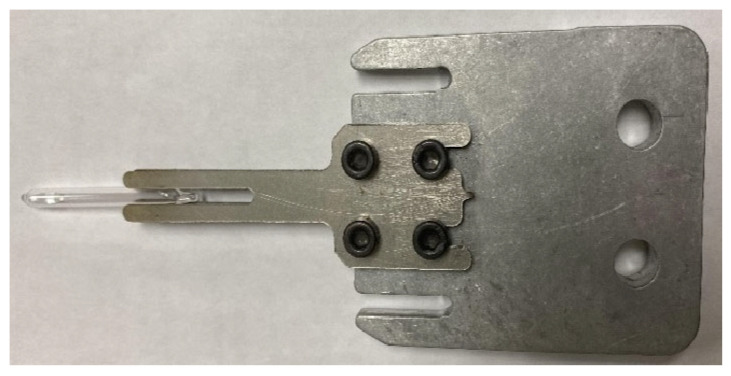
Customized ampoule holder used to position the sealed ZBLAN-containing silica ampoule on the heated stage.

The silica ampoules had a diameter of 3 mm and a length of 32 mm. After loading the ZBLAN sections and silica spacer, the ampoules were evacuated and sealed at both ends to minimize oxygen exposure and suppress convective heat transfer within the ampoule. Approximately 200 ampoules were prepared using consistent fabrication procedures. The final sealed ampoule configuration is shown in Figure 2, and the customized holder used to position the sealed ampoule on the heated stage is shown in Figure 3. Because the ZBLAN sections were obtained from the same preform batch and prepared using a consistent two-section ampoule configuration, differences observed after treatment were attributed primarily to the applied thermal and vibrational parameters.

All ampoules were prepared using the same sealing procedure and two-section sample arrangement to minimize variability between experiments. Because the ZBLAN sections were obtained from the same preform batch and processed under identical preparation conditions, differences observed after treatment were attributed primarily to the applied thermal and vibrational parameters.

### 2.3. Thermal and Vibration-Assisted Treatment Procedure

Thermal-only and vibration-assisted treatments were performed using a standardized procedure. Before each experiment, the microscope, heated stage, external temperature controller, vibration motor, cooling unit, and data acquisition system were allowed to stabilize. The heated stage was then set to the selected treatment temperature using the external temperature controller and allowed to reach the target temperature before sample insertion. For vibration-assisted tests, the vibration motor was adjusted to the desired vibration level, and the vibration response was verified using the tri-axial accelerometer before sample treatment.

After the target stage temperature was reached, the sealed ZBLAN ampoule was placed on the heated stage using the customized ampoule holder. Because direct measurement of the ZBLAN temperature inside the sealed ampoule was not feasible, the stage temperature was used as the nominal treatment temperature. Each sample was exposed to the selected thermal or combined thermal–vibration condition for 1 min to examine the early stages of crystallization under short-duration processing conditions.

Immediately after treatment, the ampoule was removed from the heated stage and rapidly quenched to room temperature using a water-saturated sponge to minimize additional crystallization during cooling. The same heating, vibration exposure, and quenching procedure was applied to all samples to ensure consistency across treatment conditions. After cooling, the ampoules were opened using a glass cutter, and the ZBLAN sections were extracted for subsequent characterization.

### 2.4. Materials Characterization

After treatment, the extracted ZBLAN sections were characterized using optical microscopy, SEM, EDS, AFM, and XRD to evaluate morphology, composition, surface roughness, and structural state. Optical microscopy was first used to assess transparency, visible crystallization, and overall morphological changes. SEM and EDS analyses were performed to examine microstructural features and compositional contrast.

**Figure 4:**
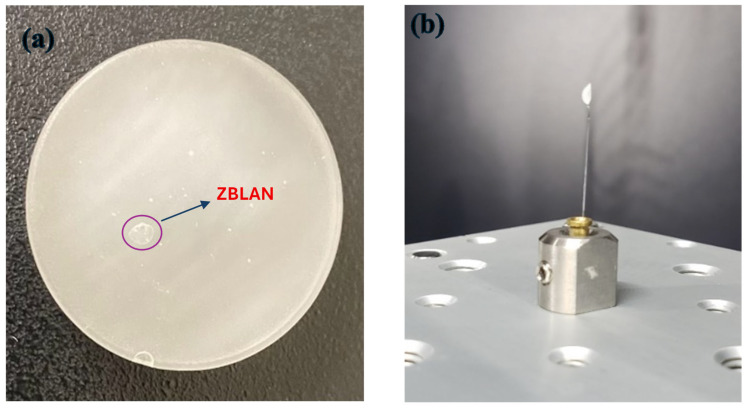
Sample mounting configurations for characterization: (**a**) epoxy-mounted ZBLAN for SEM and (**b**) ZBLAN mounted on a 100 µm glass fiber/brass pin assembly for rotating XRD.

For cross-sectional SEM analysis, selected samples were mounted in epoxy resin, cured for 72 h, and polished using successive SiC papers from 180 to 1200 grit, followed by final polishing with 6 µm and 1 µm diamond suspensions. Low-speed polishing under wet conditions was used to minimize frictional heating and reduce the possibility of polishing-induced surface crystallization, as shown in Figure 4a. AFM analysis was used to quantify surface topography and roughness changes associated with crystallization.

For XRD analysis, selected ZBLAN samples were mounted using specially designed brass pins with a 100 µm glass fiber attached to the pin. The end of the glass fiber was bonded to the ZBLAN sample using ultraviolet-curable adhesive and a three-stage micromechanical manipulator, as shown in Figure 4b. The sample/fiber/pin assembly was then mounted onto the XRD stage. During analysis, the entire assembly was rotated while a 100 µm full width at half maximum Gaussian beam analyzed the sample over a full 360° view, allowing a larger portion of each small ZBLAN specimen to be sampled. The XRD patterns were used to distinguish amorphous glassy structures from crystalline phases, with broad diffuse halos indicating amorphous structure and sharp Bragg reflections indicating crystalline phase formation.

Quantitative image and diffraction analyses were performed using ImageJ (Version 1.53) and XRD profile fitting, respectively. SEM feature coverage was estimated from selected representative SEM images by manually selecting crystal-like or morphologically distinct regions in ImageJ and calculating their area relative to the total analyzed SEM image area. Because each SEM image represents a localized sample region, the reported coverage values describe the analyzed regions rather than whole-sample crystallized-area fractions. Percent crystallinity was calculated from XRD patterns after background correction as the total integrated area of crystalline peaks divided by the total integrated area of all fitted crystalline and amorphous contributions, multiplied by 100. Therefore, the reported crystallinity values are XRD-derived estimates and should be interpreted with consideration of peak assignment and profile-fitting uncertainty.

## 3. Results and Discussion

### 3.1. Vibration Characterization

**Figure 5:**
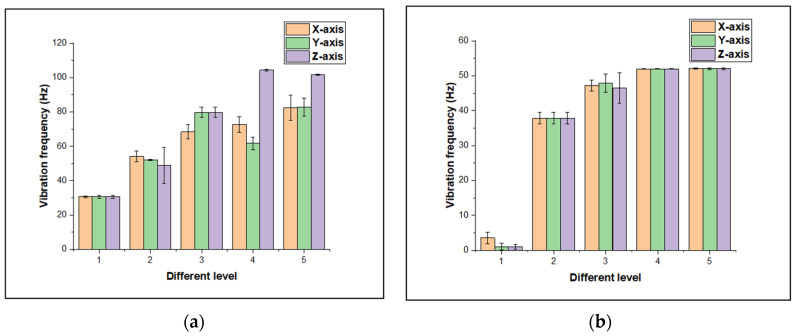
Vibration frequency value in five different levels of (**a**) high-speed vibrating motor, and (**b**) low-speed vibrating motor.

The vibration response of the testing apparatus was characterized for both the high-speed and low-speed motors, as shown in Figure 5. For the high-speed motor, the measured vibration frequency increased from approximately 30 Hz at Level 1 to approximately 50 Hz at Level 2 and then reached the highest range at Levels 3–5, with average frequencies near 70–80 Hz and maximum *z*-axis responses exceeding 100 Hz at the highest settings. The larger variation among the X, Y, and Z axes at higher levels is attributed to the structural configuration of the apparatus and damping elements within the frame. The stronger *z*-axis response further indicates that vertical vibration became more pronounced as the high-speed motor output increased.

The low-speed motor produced lower and more uniform vibration conditions. Level 1 generated only minimal vibration, below approximately 5 Hz, whereas Levels 2–5 produced frequencies in the approximate range of 35–52 Hz with comparatively small variation among the three axes. Overall, the characterization confirmed that the low-speed motor provided moderate and relatively stable vibration conditions, while the high-speed motor produced stronger and more direction-dependent vibration. These measured vibration conditions were used to correlate subsequent crystallization behavior with vibration intensity.

**Figure 6:**
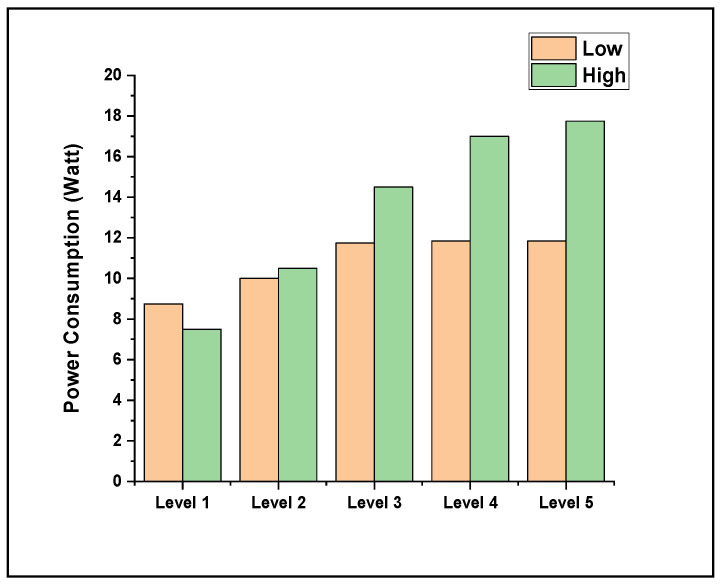
Power consumption of low-speed and high-speed vibrating motors.

Power consumption measurements further confirmed that both motors operated within their expected performance ranges, as shown in Figure 6. For the low-speed motor, power consumption increased from Level 1 to Level 3 and then remained nearly constant, indicating that the motor approached its operational limit. The high-speed motor showed a gradual increase in power consumption up to Level 4, followed by a smaller change at Level 5. This increase is attributed to the higher current required to operate the high-speed vibration motor at increasing rotational speeds. The smaller change at Level 5 suggests that the motor approached its upper operating range, where additional input resulted in only a limited increase in vibration output because of motor saturation and mechanical losses. These results are consistent with the vibration-frequency measurements and confirm that the selected motor settings provided reproducible input conditions for the thermal treatment experiments.

Following vibration characterization, ZBLAN samples were treated under temperature-only and vibration-assisted conditions. Thermal-only experiments were conducted from 250 to 400 °C to establish the baseline crystallization response. Vibration-assisted experiments were performed using all five high-speed vibration levels and selected low-speed vibration levels. Low-speed Levels 1 and 5 were excluded from the main treatment matrix because Level 1 produced negligible vibration, while Level 5 generated a response comparable to Level 4. The selected temperature range was based on the glass transition temperature, crystallization temperature, and reported crystallization behavior of ZBLAN.

### 3.2. Microscopic Analysis

**Figure 7:**
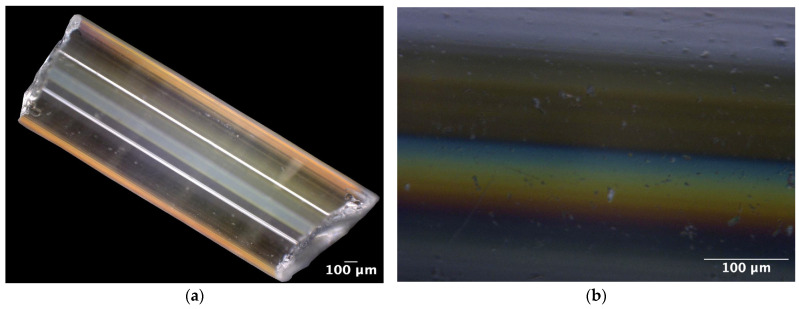
Microscopic images of ZBLAN control samples with a magnification of (**a**) 100, (**b**) 1000.

Figure 7 shows representative optical micrographs of the as-received ZBLAN samples before thermal or vibration-assisted treatment. These images were used as a baseline for comparison with treated samples. The untreated ZBLAN exhibited a transparent glassy appearance, with only minor inclusions or surface features on the micrometer scale. Because of the limited depth resolution of optical microscopy, it was not possible to determine whether these features were located on the sample surface or within the bulk. Some visible features may arise from dust, production defects, or handling-related artifacts. Therefore, SEM, EDS, AFM, and XRD analyses were used to further characterize the treated samples and support the interpretation of crystallization behavior.

**Figure 8:**
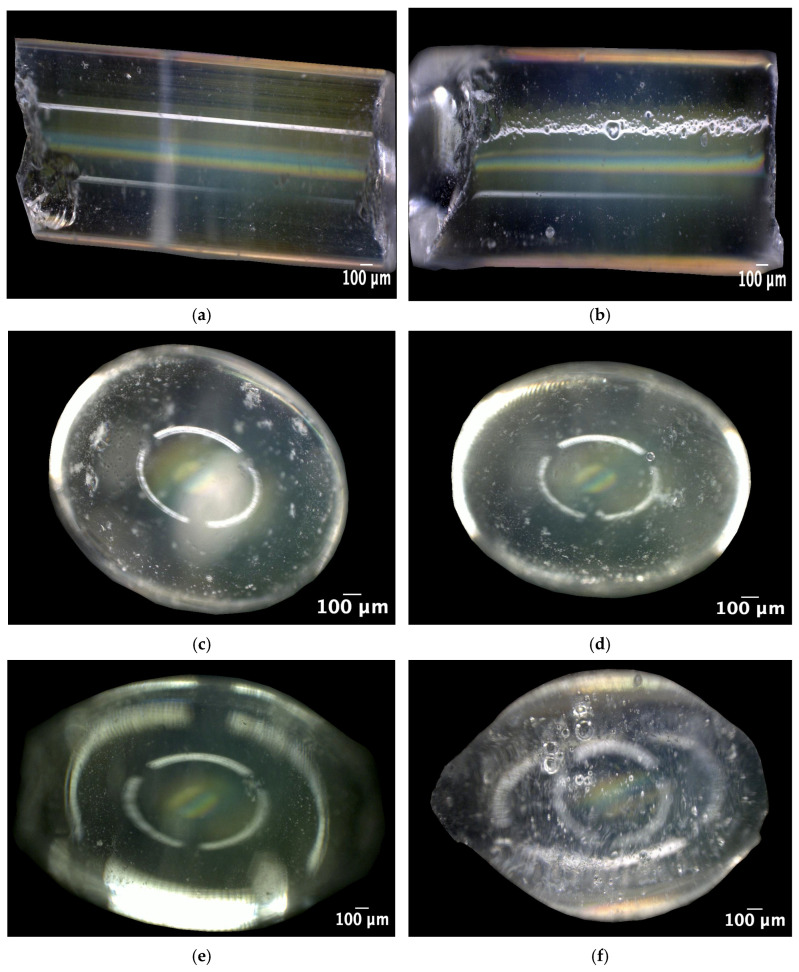

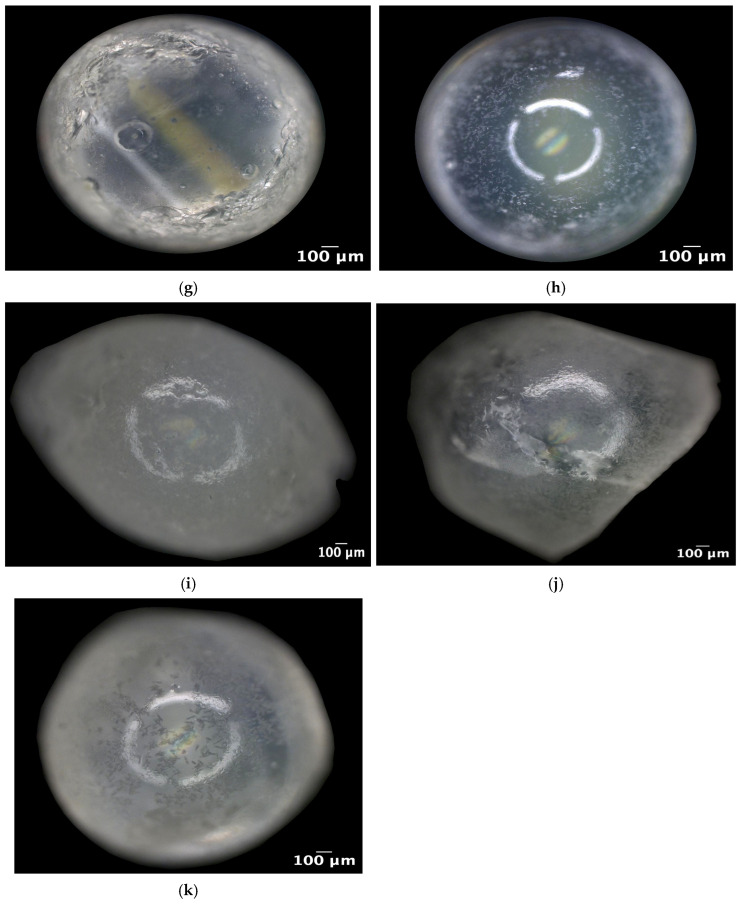
Microscopic images of ZBLAN after testing with varied temperatures: (**a**) 250 °C, (**b**) 300 °C, (**c**) 320 °C, (**d**) 330 °C, (**e**) 340 °C, (**f**) 350 °C, (**g**) 360 °C, (**h**) 370 °C, (**i**) 380 °C, (**j**) 390 °C, (**k**) 400 °C.

Similarly, Figure 8 shows optical micrographs of ZBLAN samples subjected to temperature-only treatment from 250 to 400 °C for 1 min, followed by immediate quenching. These samples were examined to establish the baseline crystallization response in the absence of mechanical vibration. From 250 to 330 °C, the samples retained a mostly transparent appearance, indicating that no significant visible crystallization occurred within this temperature range. At 340 to 350 °C, localized cloudiness and reduced transparency were observed, suggesting the onset of incipient crystallization. As the treatment temperature increased further, the samples became progressively opaquer, indicating a transition from incipient to partial crystallization. At 380 to 400 °C, pronounced opacity and visible morphological changes were observed, consistent with extensive crystallization under temperature-only treatment.

**Figure 9:**
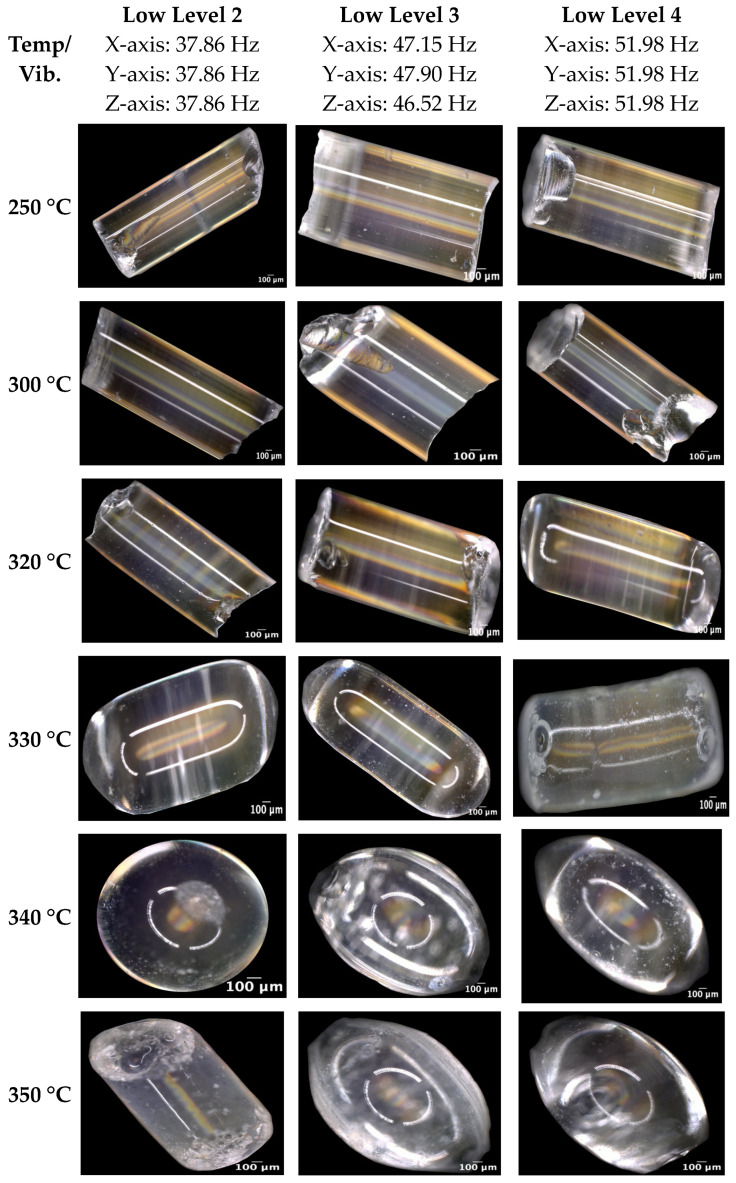

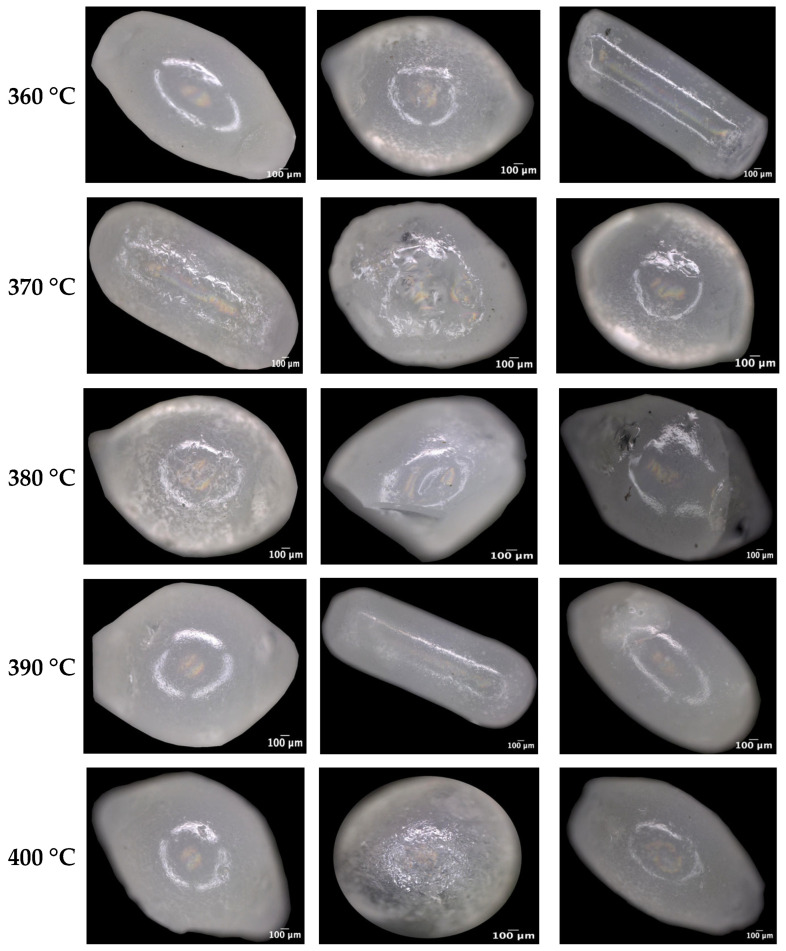
Microscopic images of ZBLAN after testing under varying temperatures and vibration frequencies using a low-speed vibrating motor.

Figure 9 shows optical micrographs of ZBLAN samples treated for 1 min under low-speed vibration at temperatures from 250 to 400 °C. Three low-speed vibration levels were evaluated: L2, L3, and L4. For L2, where the measured vibration frequency was approximately 37.86 Hz along all three axes, the samples remained mostly transparent up to 320 °C, and visible crystallization began at approximately 330 °C. For L3, with vibration frequencies of 47.15, 47.90, and 46.52 Hz along the X, Y, and Z axes, respectively, incipient crystallization was also observed near 330 °C, with more pronounced crystallization occurring above 360 °C. For L4, where the measured vibration frequency was approximately 51.98 Hz along all three axes, visible crystallization was delayed until approximately 350 °C and became more evident above 360 °C.

Overall, the low-speed vibration conditions produced crystallization behavior broadly comparable to the temperature-only baseline. Although small differences were observed among L2, L3, and L4, the apparent onset of crystallization remained within approximately 330 to 350 °C, and crystallization became more pronounced at temperatures above 360 °C. These results indicate that low-speed vibration did not substantially shift the crystallization response of ZBLAN relative to temperature-only treatment under the conditions investigated.

**Figure 10:**
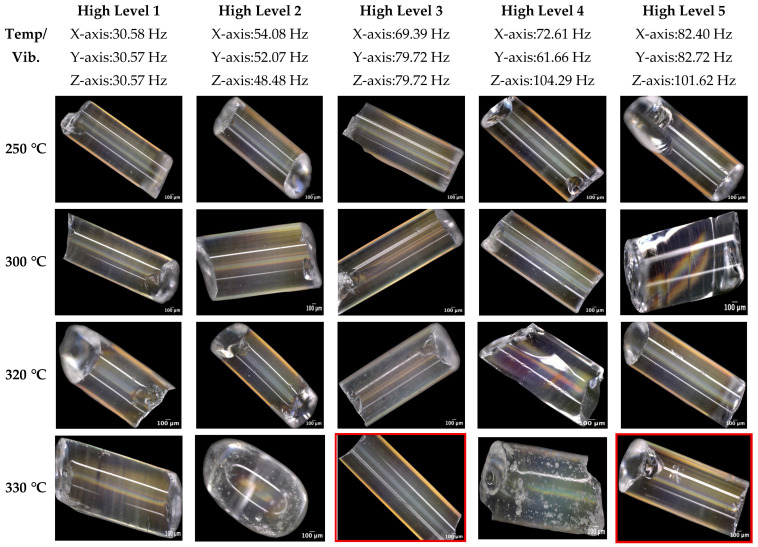

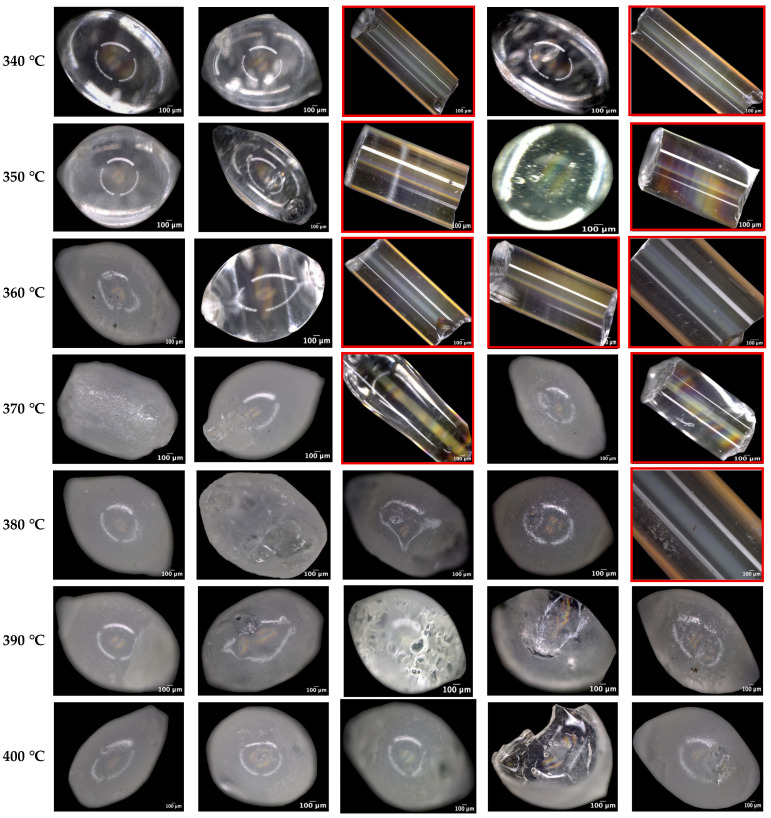
Microscopic images of ZBLAN after testing with varying temperatures and vibration frequencies using a high-speed vibrating motor.

Similarly, Figure 10 shows optical micrographs of ZBLAN samples treated for 1 min under high-speed vibration at temperatures from 250 to 400 °C. Five high-speed vibration levels were evaluated: H1, H2, H3, H4, and H5. For H1, where the measured vibration frequency was approximately 30 Hz along all three axes, the samples remained mostly transparent up to 330 °C. Incipient crystallization was observed at approximately 340 °C, and more pronounced crystallization occurred from 360 °C onward. For H2, with vibration frequencies of 54.08, 52.07, and 48.48 Hz along the X, Y, and Z axes, respectively, incipient crystallization began at approximately 330 °C, while well-developed crystallization became evident above 370 °C.

At higher vibration levels, the crystallization behavior became less monotonic. For H3, with vibration frequencies of 69.39, 79.72, and 79.72 Hz along the X, Y, and Z axes, respectively, visible crystallization was primarily observed at 380 °C, while several lower-temperature samples remained comparatively transparent. For H4, with vibration frequencies of 72.61, 61.66, and 104.29 Hz, incipient crystallization was observed at 330 to 340 °C; however, the samples treated at 350 to 360 °C showed limited visible crystallization, followed by more pronounced crystallization from 370 °C onward. For H5, with vibration frequencies of 82.40, 82.72, and 101.62 Hz, visible crystallization was delayed until approximately 390 to 400 °C.

The red-boxed samples in Figure 10 correspond to high-vibration conditions that deviated from the expected temperature-dependent crystallization trend. In these cases, several samples treated at H3, H4, and H5 remained comparatively transparent at temperatures where crystallization was otherwise anticipated. This behavior indicates that high vibration does not produce a simple monotonic increase in crystallization. Instead, excessive vibration appears to introduce a competing effect associated with sample movement inside the evacuated ampoule.

Because the ZBLAN sections were sealed inside the ampoule and direct sample-temperature measurement was not feasible, the stage temperature was used as the nominal treatment temperature. Under high-vibration conditions, movement of the ZBLAN section may intermittently reduce physical contact with the ampoule wall, thereby decreasing conductive heat transfer during the short 1 min exposure. As a result, the effective sample temperature may be lower than the nominal stage temperature, which can delay or suppress crystallization. This effect may explain why some high-vibration samples remained comparatively transparent at temperatures where crystallization was otherwise expected. Therefore, the red-boxed samples are interpreted as evidence that vibration can influence crystallization through two competing mechanisms: mechanical excitation may promote nucleation and crystal growth, whereas excessive sample motion may reduce thermal coupling and limit effective heating. This interpretation highlights that crystallization under vibration-assisted treatment is controlled not only by vibration frequency and nominal temperature, but also by sample stability and thermal contact during short-duration exposure. Further quantitative evaluation of this coupled vibration–thermal contact effect is the subject of a follow-up study.

### 3.3. Scanning Electron Microscopy (SEM) Analysis

**Figure 11:**
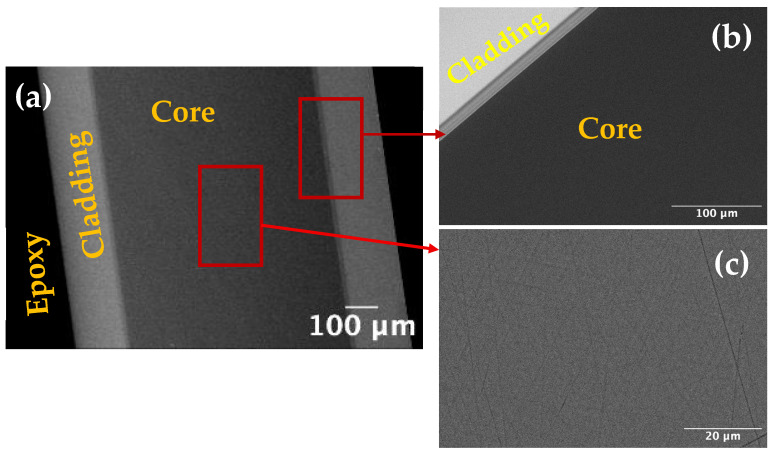
SEM images of ZBLAN showing (**a**) the core region sandwiched between cladding layers, (**b**) the core region with the surrounding cladding layer, and (**c**) the cladding layer alone.

SEM analysis was performed to evaluate the microstructural changes associated with thermal and vibration-assisted treatment of ZBLAN. Figure 11 shows backscattered SEM images of the as-received ZBLAN preform, revealing two distinct regions corresponding to the HBLAN cladding and ZBLAN core. The cladding layer exhibited an average thickness of approximately 174.5 µm, while the core region had an average width of approximately 653.74 µm. A narrow transition region was observed between the core and cladding, indicating a gradual interface between the two glass regions rather than a separate layer.

The contrast variation observed between the core and cladding regions is attributed primarily to compositional differences within the ZBLAN/HBLAN structure. Since the SEM images were acquired in backscattered electron mode, regions containing elements with higher atomic number and higher density generally produce stronger backscattered electron signals and therefore appear brighter. In the as-received preform, the HBLAN cladding appears brighter than the ZBLAN core because the cladding contains hafnium (Hf) in place of zirconium (Zr), which is present in the core. Hafnium has a higher atomic number (Z = 72) than zirconium (Z = 40) and also has a much higher density, approximately 13.31 g/cm^3^ compared with approximately 6.52 g/cm^3^ for zirconium. Therefore, the Hf-containing HBLAN cladding produces a stronger backscattered electron signal and appears brighter than the Zr-containing ZBLAN core in the SEM images [15,16].

The apparent identification and width of the transition region depend on the field of view and the distance from the core–cladding interface. When regions far from the interface are examined, the image primarily represents either the ZBLAN core or the HBLAN cladding. In contrast, when the analyzed region includes both the core and cladding, the transition zone becomes visible at the interface. Therefore, this feature is interpreted as a gradual interfacial region showing compositional continuity between the ZBLAN core and HBLAN cladding, with no visible gap, crack, or delamination.

**Figure 12:**
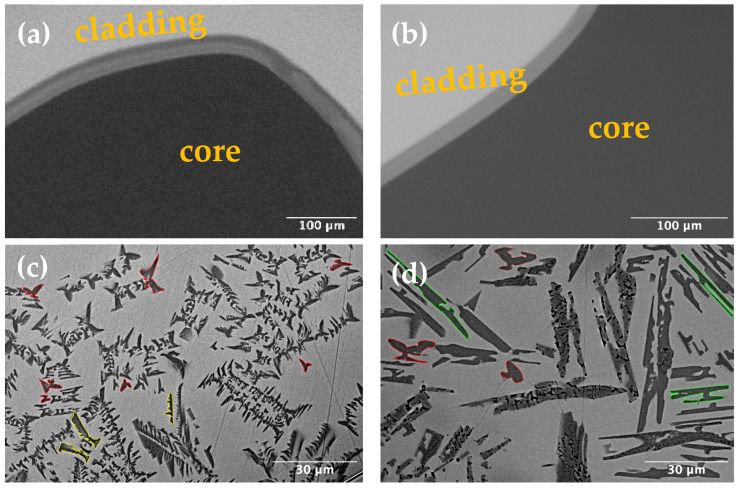
SEM images of ZBLAN while treating with different temperatures: (**a**) 320 °C, (**b**) 340 °C, (**c**) 390 °C, and (**d**) 400 °C.

SEM images of ZBLAN samples treated at different temperatures are shown in Figure 12. At 320 °C and 340 °C, no surface crystallization was observed, where the two distinct layers, core and cladding, remained clearly visible as shown in Figure 12a and Figure 12b respectively.

At a temperature of 390 °C, multiple crystals appeared on the surface of ZBLAN, as depicted in Figure 12c. The surface exhibited two distinct types of crystals: dark needle-shaped crystals (highlighted in green) and bow-tie-shaped crystals (highlighted in red). These crystals collectively occupied 33.92% of the SEM image area, which measured 3641.192 µm^2^, with the largest crystal measuring 566.934 µm^2^.

At a temperature of 400 °C, distinctive crystals of varying shapes and sizes formed on the surface of ZBLAN, as illustrated in Figure 12d. Two prominent morphologies were observed: bow-tie-shaped crystals (highlighted in red) and feather-like crystals (highlighted in yellow). These crystals collectively covered 22.04% of the SEM image area, which measured 2370.257 µm^2^. Among the observed crystals, the largest had an area of 113.031 µm^2^.

Building on the temperature-only observations, SEM analysis was also performed on selected ZBLAN samples subjected to combined thermal and vibrational treatment. The objective was to examine how vibration conditions and treatment temperature influence the morphology, distribution, and extent of crystallized surface features. The reported area fractions represent the percentage of crystallized features within the analyzed SEM image regions and should not be interpreted as bulk crystallinity.

**Figure 13:**
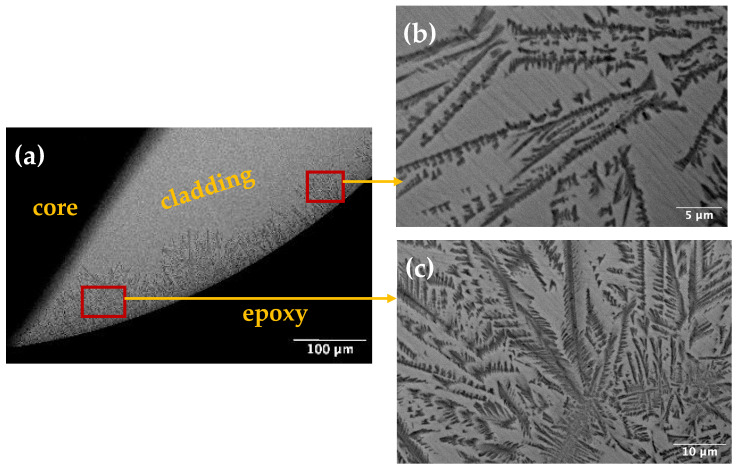
SEM images of ZBLAN treated at L3_360 under low-speed vibration, showing crystallization morphologies in (**a**) the core-cladding region, with magnified vies of selected cladding regions in (**b**) the upper-right area and (**c**) the lower-left area.

Figure 13 shows the ZBLAN sample treated at L3_360. Crystallized features were primarily observed along the cladding edge, while the core and surrounding regions remained comparatively smooth. Higher-magnification images revealed two distinct morphologies. In one region, dark needle-like features covered 26.99% of the analyzed SEM image area, corresponding to a total feature area of 235.98 µm^2^, with the largest feature measuring 27.63 µm^2^. In another region, feather-like features occupied 30.05% of the analyzed area, corresponding to 812.19 µm^2^, with the largest feature measuring 40.57 µm^2^. These results indicate that low-speed vibration at 360 °C promoted localized crystallization, particularly along the cladding boundary.

**Figure 14:**
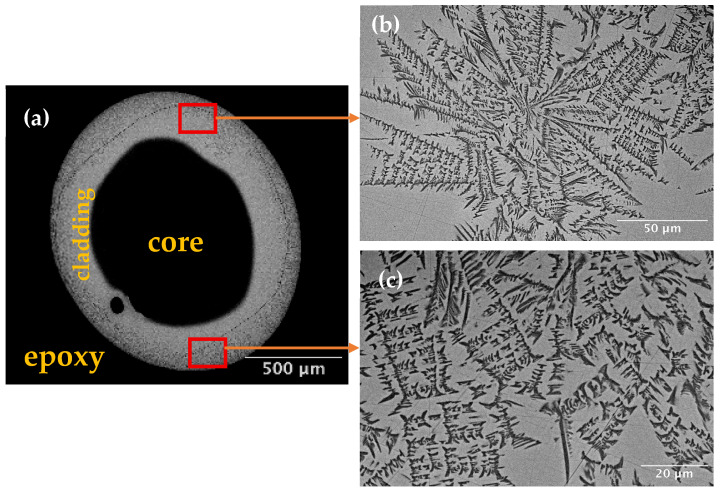
SEM images of ZBLAN treated at H1_380 under high speed vibration, showing crystallization morphologies in (**a**) the core-cladding region and magnified views of selected cladding regions in (**b**) the upper-right area and (**c**) the lower area.

Figure 14 shows the ZBLAN sample treated at H1_380. Under this high-speed vibration condition, extensive crystallized features occurred along the edge of the ZBLAN cladding layer. In the upper edge region, bow-tie-shaped features dominated, with a smaller contribution from dark needle-like features. These features covered 24.44% of the analyzed SEM image area, corresponding to a total feature area of 6172.88 µm^2^, with the largest individual feature measuring 162.21 µm^2^. In a second edge region, feather-like features were observed together with bow-tie-shaped and dark needle-like morphologies. These features covered 25.21% of the analyzed area, corresponding to 1877.02 µm^2^, with the largest feature measuring 47.98 µm^2^. The coexistence of multiple morphologies along the cladding edge suggests heterogeneous nucleation and directional growth under combined thermal and vibrational excitation.

**Figure 15:**
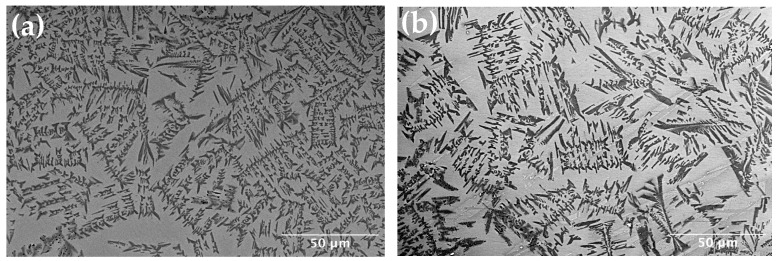
SEM images of ZBLAN treated at (**a**) H2_370 and (**b**) H3_390, showing representative high-speed vibration-assisted crystallization morphologies.

Figure 15 compares ZBLAN samples treated at H2_370 and H3_390. For H2_370, bow-tie-shaped features, dark needle-like features, and a small number of feather-like morphologies were observed. In one analyzed region, the crystallized features covered 19.61% of the SEM image area, corresponding to a total area of 2109.36 µm^2^, with the largest feature measuring 1010.12 µm^2^. In another region, dark needle-like features were more abundant and were accompanied by bow-tie-shaped and feather-like morphologies. These features occupied 29.06% of the analyzed area, corresponding to 7343.15 µm^2^, with the largest feature measuring 297.84 µm^2^. For H3_390, the surface exhibited a denser and more broadly distributed crystallized morphology dominated by dark needle-like and bow-tie-shaped features, with only a small fraction of feather-like features. The crystallized features covered 26.58% of the SEM image area, corresponding to 6767.99 µm^2^, with the largest feature measuring 313.25 µm^2^.

Quantitative SEM image-analysis results for these representative vibration-treated samples are summarized in Table 1. Morphology terms in Table 1 refer to crystal-like surface features observed within the analyzed SEM regions.

Overall, the SEM observations demonstrate that vibration-assisted treatment affects both the spatial distribution and morphology of crystallized regions in ZBLAN. Temperature-only treatment produced clear surface morphologies at elevated temperatures, while vibration-assisted treatment promoted localized and heterogeneous crystallization under selected thermal conditions. Low-speed vibration produced features mainly near the cladding boundary, whereas high-speed vibration generated more extensive and morphologically diverse features, including needle-like, bow-tie-shaped, and feather-like structures. This suggests that high-speed vibration had a stronger influence on crystallization behavior and may be more unfavorable for final optical quality, because widespread crystalline features can act as scattering centers and increase structural heterogeneity. However, this comparison remains qualitative, since optical attenuation and crystallized-area fraction were not directly measured in the present study. These observations support the optical microscopy results and show that ZBLAN crystallization is governed by the combined effects of temperature, vibration condition, and local sample geometry.

### 3.4. Energy-Dispersive X-Ray Spectroscopy (EDS) Analysis

Energy-dispersive X-ray spectroscopy (EDS) was performed to complement the SEM observations and evaluate elemental distribution in ZBLAN samples subjected to selected treatment conditions. The analysis was used to identify compositional differences between the ZBLAN core, HBLAN cladding, and crystallized surface regions. Because EDS is a localized and semi-quantitative technique, the reported elemental compositions should be interpreted as representative of the analyzed regions rather than bulk sample composition.

**Figure 16:**
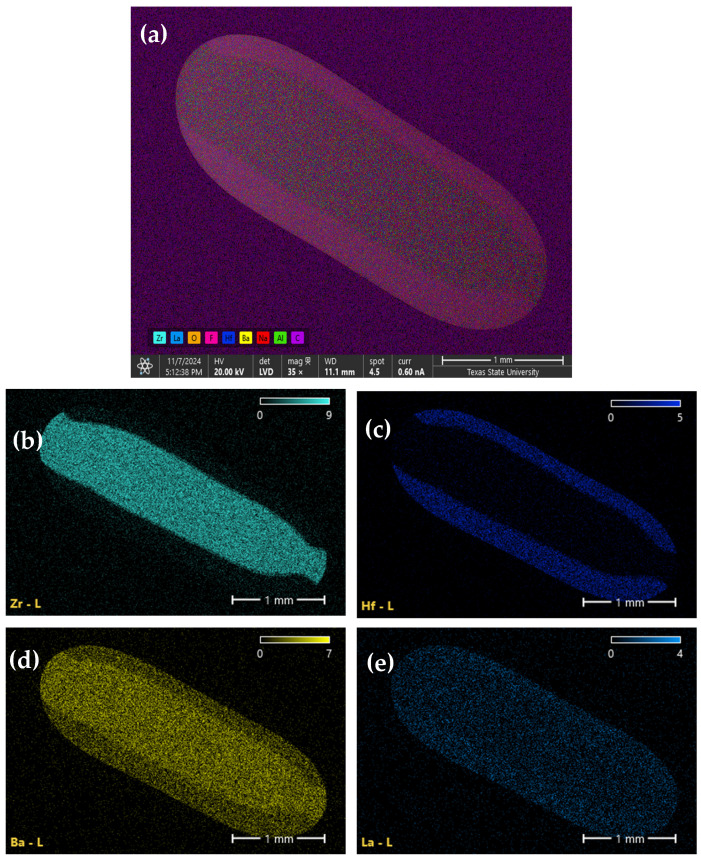

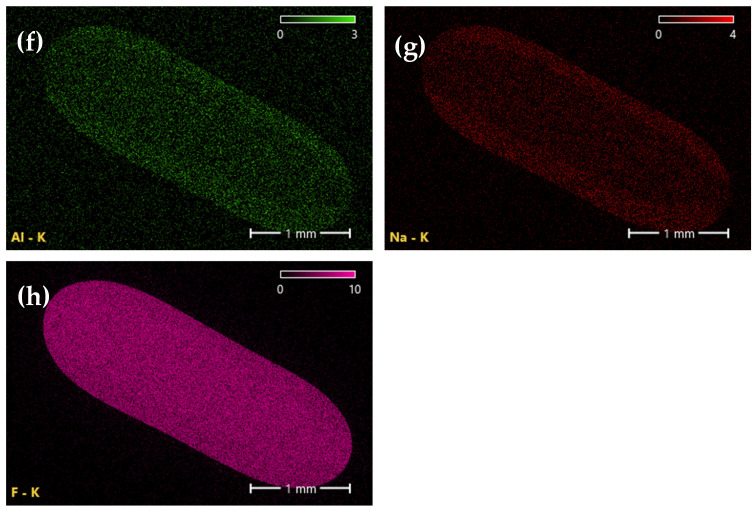
EDS elemental maps of ZBLAN treated at H1_340: (**a**) analyzed SEM region and (**b**–**h**) elemental maps of Zr, Hf, Ba, La, Al, Na, and F, respectively.

Figure 16 shows the SEM image and corresponding EDS elemental maps of the ZBLAN sample treated at H1_340. Figure 16a shows the analyzed SEM region, while Figure 16b–h show the elemental distributions of Zr, Hf, Ba, La, Al, Na, and F, respectively. The Zr signal was concentrated primarily in the inner core, while Hf was concentrated in the outer cladding, confirming the compositional distinction between the ZBLAN core and HBLAN cladding. Ba showed a stronger contribution in the core region, whereas La, Al, and Na were more concentrated in the cladding. In contrast, F was distributed throughout both regions, consistent with its role as the primary anion in both fluoride glass compositions.

**Figure 17:**
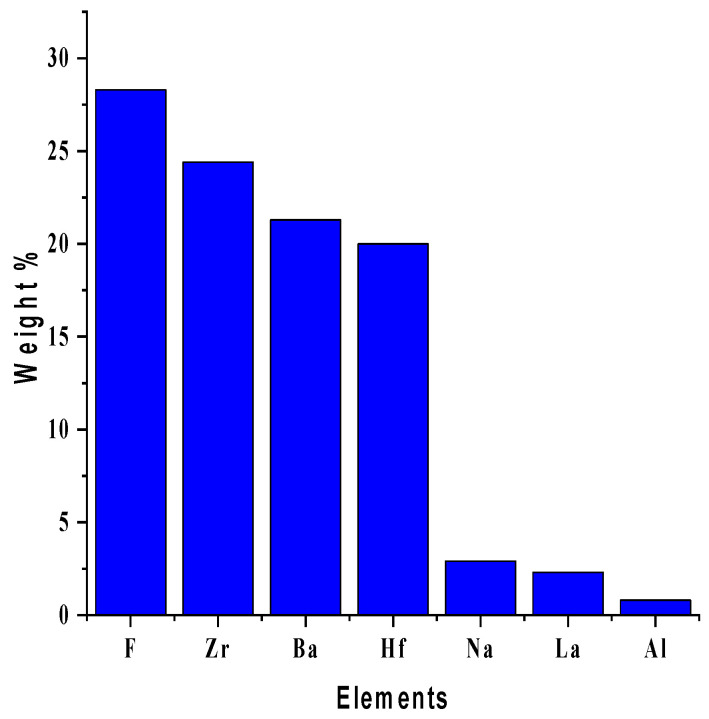
Elemental weight percentage of ZBLAN treated at H1_340.

The corresponding elemental weight percentage for the H1_340 sample is shown in Figure 17. F exhibited the highest concentration, exceeding 25 wt.%, reflecting its abundance in both the core and cladding glass networks. Zr, associated mainly with the core region, accounted for approximately 24 wt.%. Ba and Hf each contributed approximately 20 wt.%, while Na, La, and Al were present at lower concentrations, each below 5 wt.%. These results confirm that the H1_340 sample retained the expected core–cladding elemental distribution, with no strong evidence of extensive compositional redistribution within the analyzed region.

**Figure 18:**
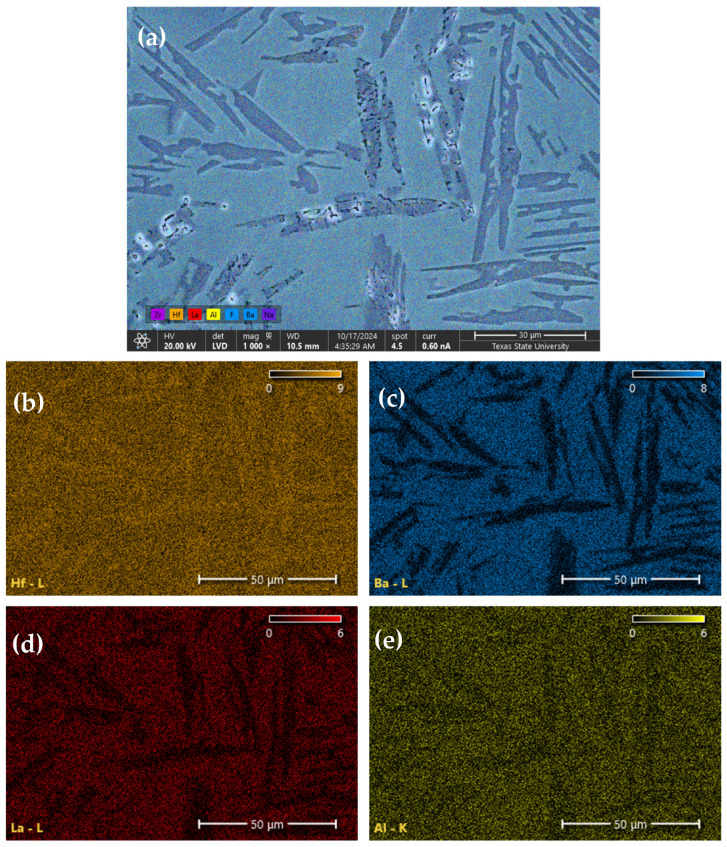

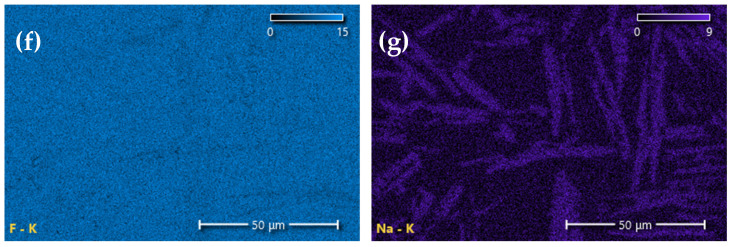
EDS elemental maps of ZBLAN thermally treated at 390 °C: (**a**) analyzed crystallized region and (**b**–**g**) elemental maps of Hf, Ba, La, Al, F, and Na, respectively.

Figure 18 presents the SEM image and corresponding EDS elemental maps of the ZBLAN sample thermally treated at 390 °C, where pronounced surface crystallization was observed within the cladding region. Figure 18a shows the analyzed crystallized region, while Figure 18b–g show the elemental distributions of Zr, Hf, Ba, La, Al, Na, and F, respectively. In this region, Zr was not detected, indicating that the analyzed crystallized features were associated primarily with the HBLAN cladding rather than the ZBLAN core. The EDS maps showed strong Hf enrichment within the crystallized surface features, while F remained broadly distributed across the analyzed region. Ba and La were not strongly associated with the selected crystal surfaces, whereas Al was only weakly detected. Na showed locally elevated intensity, suggesting possible compositional variation within the crystallized cladding region.

**Figure 19:**
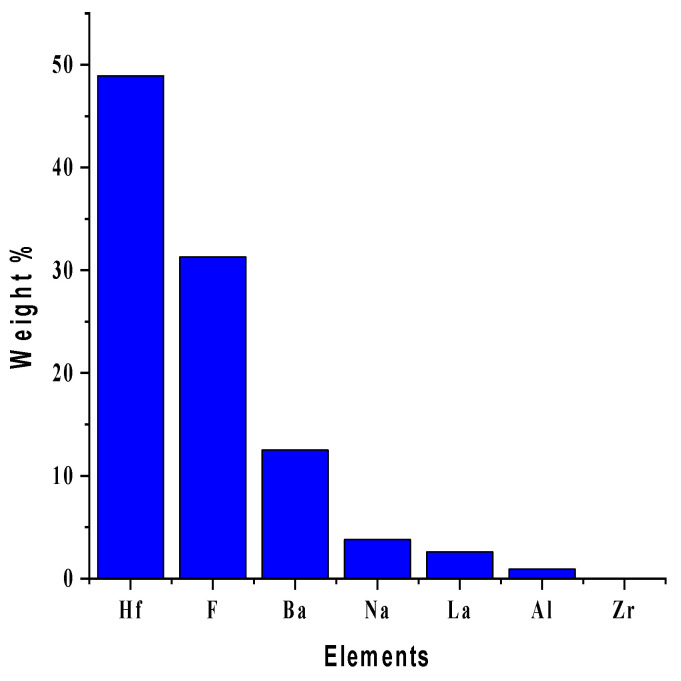
Elemental weight percentage of ZBLAN thermally treated at 390 °C.

The elemental weight percentage for the 390 °C sample is shown in Figure 19. Hf exhibited the highest concentration at nearly 50 wt.%, followed by F at approximately 30 wt.%. Ba accounted for approximately 10 wt.%, while Na, La, and Al each remained below 5 wt.%. No detectable Zr was observed in the analyzed region. This composition confirms that the crystallized surface features formed predominantly within the Hf-rich cladding layer and supports the SEM interpretation that contrast differences in the treated samples arise from local compositional variations associated with crystallization.

Overall, the EDS results confirm the expected compositional separation between the ZBLAN core and HBLAN cladding and provide chemical evidence that the crystallized surface features observed by SEM were primarily associated with the Hf-rich cladding region. The strong Hf and F signals, absence of Zr in the crystallized cladding features, and localized variations in Ba, Na, La, and Al indicate that the analyzed crystallized regions are compositionally distinct from the ZBLAN core. These findings support the SEM observations and provide a compositional basis for interpreting the bright and dark contrast variations observed in the backscattered SEM images.

### 3.5. Atomic Force Microscopy (AFM) Analysis

**Figure 20:**
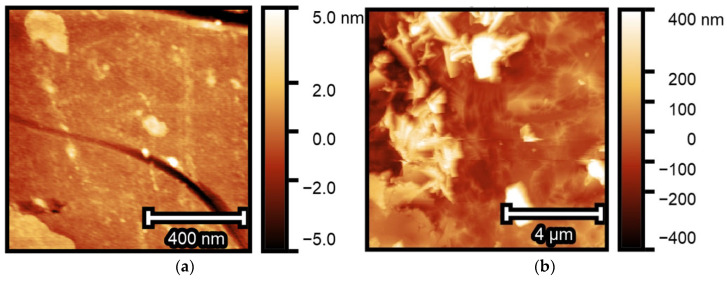
AFM images of ZBLAN: (**a**) as-received sample and (**b**) sample treated at L3_390.

Atomic force microscopy (AFM) was used to evaluate nanoscale surface topography and roughness changes associated with ZBLAN treatment. Figure 20 compares AFM images of the as-received ZBLAN sample and the sample treated at L3_390. The as-received sample exhibited a relatively smooth surface, with a root mean square (RMS) roughness of 1.168 nm, consistent with the untreated glassy surface observed by optical microscopy and SEM.

In contrast, the L3_390 sample exhibited a substantially rougher and more heterogeneous surface morphology, with an RMS roughness of 148.2 nm. This increase in roughness indicates pronounced nanoscale height variation and significant surface modification after combined thermal and vibration-assisted treatment. The rougher surface is attributed to the presence of crystal-like surface features and associated grain-boundary-like or topographic discontinuities, which increase surface height variation compared with the smooth amorphous glass surface. Therefore, the AFM results support the interpretation that crystallization is accompanied by increased surface roughness.

Overall, the AFM results provide quantitative evidence of treatment-induced surface roughening in ZBLAN. The increase in RMS roughness from 1.168 nm in the as-received sample to 148.2 nm after L3_390 treatment demonstrates that the combined thermal and vibrational condition produced pronounced nanoscale topographic changes. Since the treated sample was not fully crystallized and contained localized surface features, the AFM measurements should be interpreted as local topographic measurements rather than as a representation of the entire bulk sample. The AFM scans were performed over limited scan areas, including 400 nm and 4 µm fields of view; therefore, the reported RMS roughness values reflect the roughness of the analyzed regions and cannot be assumed to represent the whole sample surface or bulk material.

### 3.6. X-Ray Diffraction (XRD) Analysis

**Figure 21:**
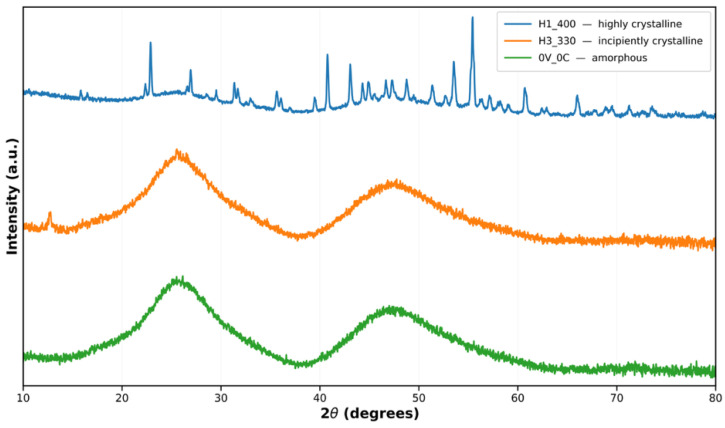
Comparative XRD patterns of 0V_0C, H3_330, and H1_400 ZBLAN samples, showing amorphous, incipiently crystalline, and highly crystalline structures, respectively.

XRD analysis was performed to compare the structural state of selected ZBLAN samples representing amorphous, incipiently crystalline, and highly crystalline conditions. As shown in Figure 21, the untreated 0V_0C sample exhibited broad diffuse halos without sharp Bragg reflections, confirming its predominantly amorphous glassy structure. This interpretation is consistent with standard XRD analysis of glass systems, where amorphous materials exhibit broad diffuse scattering rather than well-defined crystalline reflections. The H3_330 sample displayed a similar amorphous background, but with a small number of weak diffraction peaks superimposed on the diffuse halos. Therefore, H3_330 is best described as predominantly amorphous with incipient crystallization rather than as a fully crystallized sample.

In contrast, the H1_400 sample exhibited numerous sharp Bragg reflections superimposed on the amorphous background, confirming substantial crystalline phase formation under the H1_400 treatment condition. Quantitative XRD analysis indicated 0% crystallinity for 0V_0C, approximately 2.9% for the weakly crystallized H3 condition, and approximately 39.8% for H1_400. Because XRD crystallinity estimation depends on background modeling and assignment of broad peaks as amorphous or crystalline, the reported crystallinity values should be interpreted as semi-quantitative. The resolved crystalline phases in H1_400 were reported to have crystallite sizes greater than 300 nm, whereas the weakly crystallized H3 condition showed a much smaller average crystallite size of 9.9 ± 8.5 nm. Together with the crystallinity values, these results demonstrate a structural progression from amorphous ZBLAN to incipient crystallization and then to substantial crystallization.

The H1_400 XRD pattern also showed evidence of texture or preferred orientation, as several high-angle reflections exhibited higher-than-expected relative intensities, while some lower-angle reflections were comparatively weak or absent. In XRD analysis, the relative intensity of a diffraction peak depends not only on the presence of a crystalline phase, but also on factors such as the structure factor, multiplicity, Lorentz-polarization factor, absorption, crystallite size, phase fraction, and crystallite orientation distribution. Standard reference patterns generally assume a statistically random crystallite orientation, as obtained from finely ground powder specimens. In contrast, the present samples were analyzed as intact treated glass sections rather than powdered samples. Therefore, crystallized regions that formed locally at surfaces, interfaces, and thermally affected regions may have an anisotropic orientation distribution. Crystal-like morphologies observed by SEM, including needle-like, bow-tie-shaped, and feather-like features, may preferentially expose or align certain crystallographic planes relative to the sample surface and incident X-ray beam. As a result, favorably oriented planes can produce enhanced diffraction intensities, whereas other expected reflections may be suppressed or absent. Vibration-assisted treatment may also influence this intensity dependence indirectly by modifying local thermal contact, sample position, and directional crystal growth during the 1 min treatment. Because quantitative texture analysis, such as pole-figure measurement or Rietveld refinement with preferred-orientation correction, was not performed in this study, Figure 21 should be interpreted primarily as a structural comparison among the selected samples. Accordingly, XRD peak positions were used mainly for phase identification, while relative peak intensities were interpreted qualitatively rather than as quantitative indicators of phase fraction or randomly oriented powder-like crystallinity.

**Figure 22:**
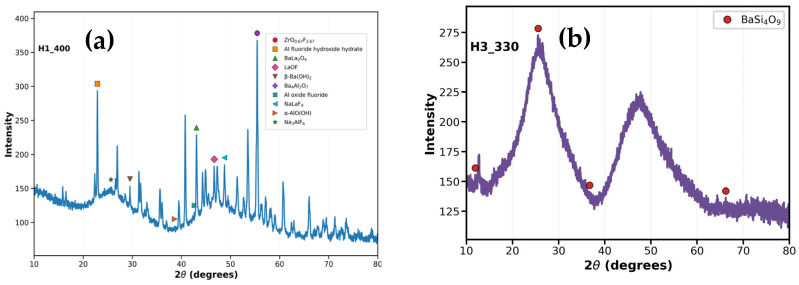
XRD phase assignments for selected ZBLAN samples: (**a**) H1_400, showing representative reflections from crystalline phases identified by XRD analysis, and (**b**) H3_330, showing low-intensity reflections associated with BaSi_4_O_9_.

Figure 22 presents the representative XRD phase assignments for the crystallized and incipiently crystallized ZBLAN samples. In Figure 22a, the H1_400 sample shows multiple sharp diffraction peaks, confirming substantial crystalline phase formation. Selected diagnostic peaks were assigned to crystalline phases identified by XRD analysis, including zirconium oxide fluoride, aluminum fluoride hydroxide hydrate, barium lanthanum oxide, lanthanum oxide fluoride, barium hydroxide, barium aluminum oxide, aluminum oxide fluoride, sodium lanthanum fluoride, aluminum oxide hydroxide, and cryolite. Because the H1_400 pattern showed preferred orientation, the relative peak intensities should be interpreted cautiously and should not be treated as those from a randomly oriented crystalline powder. Therefore, Figure 22a is used primarily to show representative phase assignments rather than quantitative phase fractions.

Figure 22b shows the H3_330 sample, which was dominated by a broad amorphous background with only a small number of low-intensity reflections. These weak reflections were associated with BaSi_4_O_9_, indicating incipient crystallization within an otherwise predominantly amorphous glass. Compared with H1_400, the reduced number and lower intensity of crystalline reflections in H3_330 confirm that crystallization was limited and at an early stage. Overall, Figure 22 supports the structural comparison shown in Figure 21 by demonstrating that H1_400 underwent substantial crystallization, whereas H3_330 remained largely amorphous with only limited crystalline development.

## 4. Conclusions

This study examined how thermal and vibration-assisted treatment influences crystallization behavior in ZBLAN glass. The main conclusions are as follows:

1. Temperature remains the primary factor controlling crystallization in ZBLAN. Increasing treatment temperature promoted progressive morphological and structural changes.
2. Vibration-assisted treatment modified the crystallization response. Low-speed vibration produced more localized features, while high-speed vibration generated broader and more morphologically diverse surface changes.
3. SEM/EDS, AFM, and XRD analyses confirmed that vibration-assisted treatment can promote surface modification, increased roughness, and structural evolution from amorphous to crystalline states under selected conditions.
4. The crystallization response was not governed by vibration frequency alone. Local sample geometry, thermal contact, and possible preferred orientation of crystallized regions also influenced the observed behavior.
5. From a practical fabrication perspective, vibration should be carefully controlled during ZBLAN fiber processing, especially within the crystallization-sensitive temperature range between the glass transition temperature (T_g_) and crystallization onset temperature (T_x_). Minimizing unnecessary mechanical vibration, maintaining stable thermal contact, and monitoring vibration exposure together with temperature will help preserve the amorphous structure and reduce crystallization-related defects.

Overall, these findings show that mechanical vibration is an important processing variable in ZBLAN fabrication and should be considered when developing terrestrial and microgravity-relevant fiber manufacturing systems. In particular, high-vibration conditions should be minimized or damped to suppress possible crystal formation and support the fabrication of low-loss amorphous ZBLAN fibers.

## Figures and Tables

**Table 1:** Quantitative analysis of SEM images from representative vibration-treated ZBLAN samples.

Sample Condition	Dominant Morphology	Feature Coverage (%)	Total Feature Area (µm^2^)	Largest Feature Area (µm^2^)
L3_360, Region I	Needle	26.99	235.98	27.63
L3_360, Region II	Feather	30.05	812.19	40.57
H1_380, Region I	Bow-tie + Needle	24.44	6172.88	162.21
H1_380, Region II	Feather + Bowtie + Needle	25.21	1877.02	47.98
H2_370, Region I	Bow-tie + Needle + Feather	19.61	2109.36	1010.12
H2_370, Region II	Needle + Bowtie + Feather	29.06	7343.15	297.84
H3_390	Needle + Bowtie	26.58	6767.99	313.25

## Data Availability

The data presented in this study are available from the corresponding author upon reasonable request.
